# Prediction of in-hospital death among patients admitted to a tertiary care hospital over the first 10 years: a machine learning approach

**DOI:** 10.3389/fpubh.2025.1635708

**Published:** 2025-10-06

**Authors:** Edel Rafael Rodea-Montero, Brenda Jesús Rodríguez-Alcántar, Dagoberto Armenta-Medina

**Affiliations:** ^1^INFOTEC Centro de Investigación e Innovación en Tecnologías de la Información y Comunicación, Aguascalientes, Mexico; ^2^Department of Research, Hospital Regional de Alta Especialidad del Bajío (HRAEB), Instituto Mexicano del Seguro Social para el Bienestar (IMSS-BIENESTAR), Leon, Mexico; ^3^SECIHTI Secretaría de Ciencia, Humanidades, Tecnología e Innovación, Mexico City, Mexico

**Keywords:** in-hospital death, logistic regression, prediction, random forest, XGBoost

## Abstract

**Purpose:**

To describe the pre- and post-admission characteristics of hospitalized patients in a tertiary care hospital and to adjust machine learning models capable of predicting and identifying the factors that are associated with and have a greater prognostic value for in-hospital death.

**Materials and methods:**

This was a retrospective study based on data from patients who were discharged from a Mexican tertiary care hospital during its first 10 years of operation (2007–2016). Preadmission characteristics were analyzed using descriptive statistics. Comparison tests (Mann–Whitney *U*) and association tests (chi-square) were applied according to the absence or presence of in-hospital death. Multivariate models (logistic regression, random forest and XGBoost) were fitted. Their ROC curves were compared using the DeLong test, and performance metrics were evaluated.

**Results:**

In total, 55,253 hospital discharges were considered, only 45,011 (0–101 years) had complete data, and the rate of in-hospital death was 4.17%. In total, 70% of the data were used for training and 30% for testing. Two-to-two comparisons between areas under the curve (AUCs) revealed that XGBoost (AUC = 0.9162) outperformed logistic regression (AUC = 0.9036) and random forest (AUC = 0.8978) (*p*-value < 0.001 in both cases). XGBoost had a sensitivity of 87%, specificity of 81.3% and balanced efficiency of 84.2%. The most relevant predictive factors were medical service that performed the admission, number of conditions, origin of the outpatient consultation of the hospital, and the main condition diagnosed at admission according to the ICD-10, age, month of admission, and day of the week of admission.

**Conclusions:**

Owing to its ability to capture complex patterns, the XGBoost model makes it possible to identify patients with a relatively high risk of in-hospital death using the data available at hospital admission. This constitutes a support tool for decision-making, helping to determine which patients require closer monitoring and follow-up during their hospital stay to improve the quality of medical care.

## 1 Introduction

A hospital discharge occurs when a patient's hospitalization period ends and a hospital bed is vacated, either because of medical discharge or death. Data on hospital discharge by death can be valuable resources for hospital planning and management (1). In Mexico, the General Directorate of Health Information (DGIS, acronym in Spanish) is the operational body of the Ministry of Health (SSA, acronym in Spanish) that is responsible for generating statistics on health. Among the information subsystems that it manages is the Automated Hospital Discharge System (SAEH, acronym in Spanish) (2), which contains an accumulation of public data of approximately three million records per year (with at most 140 variables captured for each record) of patients who are hospitalized in a hospital unit of the Ministry of Health of Mexico (3, 4).

During the hospital stay of a patient, that is, from admission to discharge from the hospital, various adverse events may occur, among which death (also known as in-hospital death) stands out. The importance of this adverse event has been reflected in the international arena, where measures have been implemented for monitoring and analysis. For example, since 1986, the Health Care Financing Administration (HCFA) has incorporated the percentage of hospital mortality as a quantitative indicator to compare American hospitals (5). Hospital mortality is a widely used indicator of the quality of medical care (6), and the quantification of in-hospital deaths can be considered a measure of the effectiveness of hospital intervention (7). A high percentage of in-hospital deaths are associated with deficiencies in the quality of hospital care (8, 9).

The number of hospital discharges, both in general and with respect to those corresponding to deaths, varies according to hospital conditions and procedures. From the perspective of the analysis of data on hospital discharge, in Mexico, there are descriptive reports on hospital discharge at the national level (2) or at the regional level (10) that provide details on discharges due to deaths; these reports are similar to those described in the international literature (11–14). It is estimated that between 2% and 3% of hospital discharges result in death (3, 6). Identifying the factors that affect the likelihood of in-hospital death is essential for the construction of predictive models. There is evidence of factors that can increase the risk of in-hospital death, such as hospital admission during weekends (15, 16), increases in the number of hospitalized patients (17, 18) and increases in the volume of surgical patients (19, 20).

With respect to the prediction of the likelihood of in-hospital death during hospitalization, multivariate logistic regression models that are validated by an analysis of the area under the receiver operating characteristic (ROC) curve, are typically constructed (21). Then, a confusion matrix is created to evaluate the performance of the model by calculating various efficiency metrics, such as sensitivity, specificity, balanced efficiency, positive predictive value, negative predictive value, precision, recall and F1 score. The implementation of multivariate logistic regression models (22) to predict in-hospital death has allowed the identification of predictive factors such as the age of the patient (23–25), the sex of the patient (25, 26), whether the patient is a clinical or surgical patient (24), the patient's diagnosis (26), the type of disease (25, 26) and the presence of hypotension (27).

Today, the prediction of the in-hospital death of patients can be approached as a machine learning problem. This approach is considered an indispensable tool for revealing answers to complex questions in medicine (28). This is especially the case through the use of supervised machine learning, which is based on data labeled as the presence or absence of in-hospital death. With the current computing power, it is possible to implement prediction techniques using complex algorithms, whose predictive capacity has been shown to be superior to that resulting from logistic regression. These predictive algorithms include decision trees (29), random forest (30), neural networks (31), naive Bayes (32), vector support machines (33), and XGBoost (34).

At a global level, various machine learning–based risk prediction models have been developed and compared to predict in-hospital death across diverse populations and clinical settings, including the United States (35), Europe (36), Asia (37), Oceania (38), and Africa (39). These studies highlight the widespread adoption and effectiveness of supervised algorithms for early risk stratification at hospital admission, while also illustrating the variability of performance across healthcare systems and patient populations. In contrast, there is little to no evidence of similar models being applied or systematically evaluated in Latin American healthcare systems.

While previous studies have used machine learning to predict in-hospital mortality, few have specifically focused on the Mexican or Latin American healthcare context; strictly based on administrative and preliminary diagnostic data available at the point of admission, which is crucial for early, low-cost risk stratification; and systematically compared a range of advanced models, including XGBoost, on such a large, decade-long cohort.

The aim of this study was to describe the pre- and post-admission characteristics of hospitalized patients in a tertiary care hospital and to develop a risk prediction model for in-hospital death based on machine learning. By systematically comparing algorithms such as logistic regression, random forest, and XGBoost, the study identified the best-performing model and proposed it as a robust risk index tailored to the Mexican healthcare context, which provides a foundation for future integration into clinical workflows as a decision-support tool.

## 2 Materials and methods

### 2.1 Patients

We conducted a retrospective study of all the records of patients (*n* = 55,253) who were discharged from a Mexican tertiary care hospital (HRAEB) during its first 10 years of operation (April 2007 to December 2016). The dataset constitutes a secondary base and was obtained from the national registries of the Automated Subsystem of Hospital Expenditures (SAEH, acronym in Spanish) operated by the General Directorate of Health Information of the Secretary of Health of Mexico (DGIS, acronym in Spanish) (3). The SAEH is a system that compiles data on hospital discharges from Mexican hospitals as a primary source. The data included 16 preadmission characteristics of the patients (age, sex, state of residence, municipality of residence, has healthcare entitlement, day of the week of admission, month of admission, first hospitalization, origin of outpatient consultation of the Hospital, presence of injury, initial preadmission reference diagnoses according to the ICD-10, medical service that performed the admission, main condition diagnosed at admission according to the ICD-10, initial preadmission reference diagnoses equal to the main condition diagnosed at admission, the ICD-10 group of initial preadmission reference diagnoses equal to the ICD-10 group of the main condition diagnosed at admission, and number of comorbidities) that were collected.

In addition, data on 12 post-admission characteristics of the patients (number of diagnostic, surgical or therapeutic procedures performed, number of procedures under general anesthesia, length of stay (days), length of stay > 48 h, length of stay ≥7 days, length of stay ≥14 days, nosocomial infection, day of the week of discharge, month of discharge, medical service that performed the discharge, discharge service equal to admission service, and in-hospital death) were collected.

For the analysis, only the dataset of patients with complete records (*n* = 45,011) was considered, among whom 4.17% (1,879/45,011) experienced in-hospital death. The dataset was divided into two parts by using the train_test_split function of the R statistical package “rsample: General Resampling Infrastructure” (40). With 70% of the records (*n* = 31,507), a set was formed for the construction of models (training dataset), and with 30% of the records (*n* = 13,504), a set was formed to evaluate the models (test dataset).

### 2.2 Statistical analysis

All the data were analyzed using the R programming language (version 4.3.3, R Core Team, Vienna, Austria) (41). Initially, hospital discharges were counted in general and by reason of in-hospital death per year. Next, considering the dataset of the patients with complete records, descriptive statistics of the preadmission and post-admission characteristics of the patients in general were calculated and grouped by the absence or presence of in-hospital death. These characteristics were subjected to a comparison or association search via Mann–Whitney *U* tests (42) or chi–square tests (43), depending on the type of variable being analyzed. To predict in-hospital death and through the use of the training dataset, multivariate models were fitted [logistic regression (22), random forest (30) and XGBoost (44)] considering the preadmission characteristics of the patients as predictive variables, and the importance of the variables for each model was determined. All this was accomplished with the statistical packages R “caret: Classification and Regression Training” (45), “RandomForest: Breiman and Cutler's Random Forests for Classification and Regression” (46), and “xgboost: Extreme Gradient Boosting” (47).

For each case, the fit of the model was evaluated using the Hosmer–Lemeshow test (48). The coefficient of determination [Nagelkerke's R2 (49)] or the analysis of the area under the ROC curve (21) and optimal cutoff points were estimated, as were efficiency metrics (specificity, sensitivity, balanced accuracy, negative predictive value, positive predictive value, precision, recall and F1 score) on the basis of the confusion matrices generated with each model for both the training and test datasets. The optimal cutoff point in each case was calculated as the minimum value of the square root of [(1-sensitivity)2 + (1-specificity)2], which reflects a better accuracy due to a smaller distance to the point (0, 1) of the respective ROC curve in each case (50). The ROC curves of each multivariate model that resulted when the test dataset was considered were compared two by two through the implementation of DeLong tests (51). In all cases, 95% confidence intervals were constructed, and a level of significance of alpha = 0.05 was used in all tests. This study is consistent with “the Transparent Reporting of Multivariate Predictive Models for Individual Prognosis or Diagnosis (TRIPOD): TRIPOD statement” (52).

## 3 Results

### 3.1 Hospital discharges and in-hospital deaths

From 2007 to 2016 in the Automated Hospital Discharge System of Mexico, a total of 27,426,516 hospital discharges were registered, and when all the hospitals of the Ministry of Health were considered, the percentage of in-hospital deaths was 2.15% (588,710/27,426,516). Among all hospital discharges, only 55,253 were from the selected tertiary care hospital, where 3.66% (2,025/55,253) were due to in-hospital death. Data on the total number of hospital discharges and the number of discharges due to in-hospital death at both the national and hospital selected levels per year are shown in Table 1.

**Table 1:** Hospital discharges from all hospitals of the Ministry of Health of Mexico registered in the Automated Hospital Discharge System per year from 2007 to 2016.

**Year**	**Hospital discharges of the Ministry of Health of Mexico**	**In-hospital deaths of the Ministry of Health of Mexico**	**Hospital discharges of the selected hospital**	**In-hospital deaths of the selected hospital**
2007	2,311,826	51,047	623	29
2008	2,463,847	52,192	3,045	146
2009	2,598,366	55,147	5,063	292
2010	2,634,339	53,103	5,443	257
2011	2,775,101	57,957	5,464	244
2012	2,880,606	59,765	5,884	201
2013	2,879,313	62,571	6,361	206
2014	2,959,197	64,923	6,948	189
2015	2,970,812	66,093	7,867	240
2016	2,953,109	65,912	8,555	221
Total	27,426,516	588,710	55,253	2,025

Of the 55,253 hospital discharges, 10,242 (18.54%) were excluded due to incomplete data in at least one of the 28 variables analyzed (16 pre-admission and 12 post-admission). Most variables (23 of 28) were fully complete; the five with missing data had completeness rates of 99.998% (55,252/55,253) for age, 99.982% (55,243/55,253) for sex, 99.929% (55,214/55,253) for first hospitalization, 87.069% (48,108/55,253) for medical service that performed the admission, and 94.4% (52,170/55,253) for nosocomial infection.

Among the 10,242 excluded records, 146 (1.43%) resulted in-hospital dead, indicating that data loss was not concentrated among patients with adverse outcomes. No systematic pattern of missingness related to patient characteristics or illness severity was identified, minimizing potential selection bias.

### 3.2 Preadmission characteristics

The final analysis included the data of 45,011 patients with complete records, namely, 21,580 (47.94%) women and 23,431 (52.06%) men, hospitalized in the selected hospital during the study period. The mean age (±standard deviation) of all patients at hospital admission was 33.72 ± 24.64 years, with the ages ranging from < 1 to 101 years. In terms of hospitalization, 32,374/45,011 (71.92%) patients were first-time hospitalization cases. In addition, 40,964/45,011 (91.01%) of the hospitalized patients, originated from outpatient consultations at the hospital, and 1,879/45,011 (4.17%) of the hospital discharges were in-hospital deaths.

Data on the preadmission characteristics of the patients hospitalized during the study period in general and grouped by the absence or presence of in-hospital death are detailed in Table 2. An intergroup comparison using the Mann–Whitney *U* test revealed that the age of the patients who died was significantly higher than that of those who were discharged alive (42.62 vs. 33.33 years, *p* < 0.001). Additionally, after data analysis with the chi-square test, associations between the variable presence or absence of in-hospital death and the following variables were identified: age group, residence in the state where the hospital is located, residence in the municipality where the hospital is located, has healthcare entitlement, day of the week of admission, origin of outpatient consultation of the Hospital, presence of injury (*p* < 0.001 in all cases), month of admission (*p* = 0.024) and first hospitalization (*p* = 0.027).

**Table 2:** Preadmission characteristics of hospitalized patients in the selected hospital from 2007 to 2016.

**Variable**	**Overall (*n* = 45,011)**	**In-hospital death (–) (*n* = 43,132)**	**In-hospital death (+) (*n* = 1,879)**	**Intergroup comparison**
Age, years	33.72 (24.64)	33.33 (24.51)	42.62 (25.95)	*p* < 0.001^a*^
**Age group**				
< 1 year, *n* (%)	1,948 (4.33%)	1,776 (4.12%)	172 (9.15%)	
1–4 years, *n* (%)	5,291 (11.75%)	5,181 (12.01%)	110 (5.85%)	
5–14 years, *n* (%)	6,286 (13.97%)	6,190 (14.35%)	96 (5.11%)	
15–44 years, *n* (%)	14,552 (32.33%)	14,074 (32.63%)	478 (25.44%)	
45–64 years, *n* (%)	11,076 (24.61%)	10,486 (24.31%)	590 (31.40%)	
65 years and more, *n* (%)	5,858 (13.01%)	5,425 (12.58%)	433 (23.04%)	
**Sex**				*p* = 0.187^b^
Female, *n* (%)	21,580 (47.94%)	20,651 (47.88%)	929 (49.44%)	
Male, *n* (%)	23,431 (52.06%)	22,481 (52.12%)	950 (50.56%)	
Resides in the state where the hospital is located, *n* (%)	42,846 (95.19%)	41,101 (95.29%)	1,745 (92.87%)	*p* < 0.001^b*^
Resides in the municipality where the hospital is located, *n* (%)	12,825 (28.49%)	12,423 (28.80%)	402 (21.39%)	*p* < 0.001^b*^
Has healthcare entitlement, *n* (%)	44,219 (98.24%)	42,413 (98.33%)	1,806 (96.11%)	*p* < 0.001^b*^
**Day of the week of admission**				*p* < 0.001^b^^*^
Monday, *n* (%)	11,196 (24.87%)	10,884 (25.23%)	312 (16.60%)	
Tuesday, *n* (%)	8,663 (19.25%)	8,306 (19.26%)	357 (19.00%)	
Wednesday, *n* (%)	8,432 (18.73%)	8,102 (18.78%)	330 (17.56%)	
Thursday, *n* (%)	7,067 (15.70%)	6,756 (15.66%)	311 (16.55%)	
Friday, *n* (%)	3,588 (7.97%)	3,320 (7.70%)	268 (14.26%)	
Saturday, *n* (%)	1,522 (3.38%)	1,378 (3.19%)	144 (7.66%)	
Sunday, *n* (%)	4,543 (10.09%)	4,386 (10.17%)	157 (8.36%)	
**Month of admission**				*p* = 0.024^b^^*^
January, *n* (%)	3,407 (7.57%)	3,244 (7.52%)	163 (8.67%)	
February, *n* (%)	3,485 (7.74%)	3,333 (7.73%)	152 (8.09%)	
March, *n* (%)	3,730 (8.29%)	3,584 (8.31%)	146 (7.77%)	
April, *n* (%)	3,420 (7.60%)	3,268 (7.58%)	152 (8.09%)	
May, *n* (%)	3,700 (8.22%)	3,560 (8.25%)	140 (7.45%)	
June, *n* (%)	3,902 (8.67%)	3,726 (8.64%)	176 (9.37%)	
July, *n* (%)	4,013 (8.92%)	3,864 (8.96%)	149 (7.93%)	
August, *n* (%)	4,109 (9.13%)	3,959 (9.18%)	150 (7.98%)	
September, *n* (%)	3,889 (8.64%)	3,755 (8.71%)	134 (7.13%)	
October, *n* (%)	4,289 (9.53%)	4,101 (9.51%)	188 (10.01%)	
November, *n* (%)	3,722 (8.27%)	3,551 (8.23%)	171 (9.10%)	
December, *n* (%)	3,345 (7.43%)	3,187 (7.39%)	158 (8.41%)	
First hospitalization, *n* (%)	32,374 (71.92%)	31,065 (72.02%)	1,309 (69.66%)	*p* = 0.027^b^*
Origin of outpatient consultation of the Hospital, *n* (%)	40,964 (91.01%)	40,271 (93.37%)	693 (36.88%)	*p* < 0.001^b*^
External cause (only in case of injury), *n* (%)	2,107 (4.68%)	2,057 (4.77%)	50 (2.66%)	*p* < 0.001^b*^

Unless otherwise indicated, data are presented as the mean (standard deviation).

^a^Mann–Whitney *U* test.

^b^Chi-square test.

^*^Significant *p*-value.

Table 3 shows the initial preadmission reference diagnoses according to the ICD-10 group of patients hospitalized during the study period in general and grouped by the absence or presence of in-hospital death, highlighting that the four main preadmission reference ICD-10 diagnosis groups in a descending order (approximately 60% total) were as follows: [C00-D48] Neoplasms 14,245/45,011 (31.65%), [R00-R99] Symptoms, signs and abnormal clinical findings and laboratory findings, not elsewhere classified 5,549/45,011 (12.33%), [Q00-Q99] Congenital malformations, deformities and chromosomal abnormalities 4,475/45,011 (9.94%), and [N00-N99] Diseases of the genitourinary tract 4,182/45,011 (9.29%). In addition, an association between the preadmission reference ICD-10 diagnosis group and the absence or presence of in-hospital death was identified (*p* < 0.001).

**Table 3:** Initial preadmission reference diagnoses according to the ICD-10 group of patients hospitalized in the selected hospital from 2007 to 2016.

**Variable**	**Overall (*n* = 45,011)**	**In-hospital death (–) (*n* = 43,132)**	**In-hospital death (+) (*n* = 1,879)**	**Intergroup comparison**
**Initial preadmission reference diagnoses according to the ICD-10 group**				*p* < 0.001^a*^
I.- A00-B99 Certain infectious and parasitic diseases, *n* (%)	489 (1.09%)	461 (1.07%)	28 (1.49%)	
II.- C00-D48 Neoplasms, *n* (%)	14,245 (31.65%)	13,514 (31.33%)	731 (38.90%)	
III.- D50-D89 Diseases of the blood and hematopoietic organs and other disorders that affect the mechanism of immunity, *n* (%)	723 (1.61%)	697 (1.62%)	26 (1.38%)	
IV.- E00-E90 Endocrine nutritional and metabolic diseases, *n* (%)	1,031 (2.29%)	1,010 (2.34%)	21 (1.12%)	
V.- F00-F99 Mental and behavioral disorders, *n* (%)	144 (0.32%)	139 (0.32%)	5 (0.27%)	
VI.- G00-G99 Diseases of the nervous system, *n* (%)	1,472 (3.27%)	1,434 (3.32%)	38 (2.02%)	
VII.- H00-H59 Diseases of the eye and its annexes, *n* (%)	109 (0.24%)	109 (0.25%)	0 (0.00%)	
VIII.- H60-H95 Diseases of the ear and mastoid process, *n* (%)	328 (0.73%)	327 (0.76%)	1 (0.05%)	
IX.- I00-I99 Diseases of the circulatory system, *n* (%)	3,265 (7.25%)	2,985 (6.92%)	280 (14.90%)	
X.- J00-J99 Diseases of the respiratory system, *n* (%)	1,221 (2.71%)	1,126 (2.61%)	95 (5.06%)	
XI.- K00-K93 Diseases of the digestive system, *n* (%)	2,431 (5.40%)	2,315 (5.37%)	116 (6.17%)	
XII.- L00-L99 Diseases of the skin and subcutaneous tissue, *n* (%)	153 (0.34%)	146 (0.34%)	7 (0.37%)	
XIII.- M00-M99 Diseases of the musculoskeletal system and connective tissue, *n* (%)	2,398 (5.33%)	2,371 (5.50%)	27 (1.44%)	
XIV.- N00-N99 Diseases of the genitourinary system, *n* (%)	4,182 (9.29%)	4,123 (9.56%)	59 (3.14%)	
XV.- O00-O99 Pregnancy, childbirth and puerperium, *n* (%)	1 (0.00%)	1 (0.00%)	0 (0.00%)	
XVI.- P00-P96 Certain conditions originating in the perinatal period, *n* (%)	60 (0.13%)	59 (0.14%)	1 (0.05%)	
XVII.- Q00-Q99 Congenital malformations, deformities and chromosomal abnormalities, *n* (%)	4,475 (9.94%)	4,318 (10.01%)	157 (8.36%)	
XVIII.- R00-R99 Symptoms, signs and abnormal clinical findings and Laboratory, not elsewhere classified, *n* (%)	5,549 (12.33%)	5,297 (12.28%)	252 (13.41%)	
XIX.- S00-T98 Trauma, poisoning and some other consequences of external cause, *n* (%)	1,555 (3.45%)	1,530 (3.55%)	25 (1.33%)	
XX.- V01-Y98 Extreme causes of morbidity and mortality, *n* (%)	0 (0.00%)	0 (0.00%)	0 (0.00%)	
XXI.- Z00-Z99 Factors that influence the state of health and contact with health services, *n* (%)	1,180 (2.62%)	1,170 (2.71%)	10 (0.53%)	
XXII.- U00-U99 Codes for special situations, *n* (%)	0 (0.00%)	0 (0.00%)	0 (0.00%)	

^a^Chi-square test.

^*^Significant *p*-value.

As shown in Table 4, the medical services that performed the admission of patients in general are detailed and grouped according to the absence or presence of in-hospital death, highlighting that the services that contributed to at least 5.00% of the admissions were the following in a descending order: oncology, 8,107/45,011 (18.01%); urology, 2,729/45,011 (6.06%); neurosurgery, 2,605/45,011 (5.79%); pediatric oncology, 2,548/45,011 (5.66%); traumatology, 2,389/45,011 (5.31%); and cardiology, 2,275/45,011 (5.05%). In addition, an association between the medical service that performed the admission and the absence or presence of in-hospital death was identified (*p* < 0.001). The five medical services that accounted for approximately half of the in-hospital deaths (926/1,879) were oncology, 398/1,879 (21.18%); hematology, 179/1,879 (9.53%); internal medicine 173/1,879 (9.21%); neurosurgery 90/1,879 (4.79%); and cardiology, 86/1,879 (4.58%).

**Table 4:** Medical services that performed the admission of patients in the selected hospital from 2007 to 2016.

**Variable**	**Overall (n = 45,011)**	**In-hospital death (–) (*n* = 43,132)**	**In-hospital death (+) (*n* = 1,879)**	**Intergroup comparison**
**Medical service that performed the admission**				*p* < 0.001^a*^
Oncology, *n* (%)	8,107 (18.01%)	7,709 (17.87%)	398 (21.18%)	
Urology, *n* (%)	2,729 (6.06%)	2,707 (6.28%)	22 (1.17%)	
Neurosurgery, *n* (%)	2,605 (5.79%)	2,515 (5.83%)	90 (4.79%)	
Pediatric oncology, *n* (%)	2,548 (5.66%)	2,507 (5.81%)	41 (2.18%)	
Traumatology, *n* (%)	2,389 (5.31%)	2,381 (5.52%)	8 (0.43%)	
Cardiology, *n* (%)	2,275 (5.05%)	2,189 (5.08%)	86 (4.58%)	
Hematology, *n* (%)	1,899 (4.22%)	1,720 (3.99%)	179 (9.53%)	
Pediatric cardiology, *n* (%)	1,465 (3.25%)	1,436 (3.33%)	29 (1.54%)	
Pediatric urology, *n* (%)	1,387 (3.08%)	1,387 (3.22%)	0 (0.00%)	
Internal medicine, *n* (%)	1,252 (2.78%)	1,079 (2.50%)	173 (9.21%)	
Pediatric hematology, *n* (%)	1,223 (2.72%)	1,200 (2.78%)	23 (1.22%)	
Pediatrics, *n* (%)	1,030 (2.29%)	978 (2.27%)	52 (2.77%)	
Nephrology, *n* (%)	967 (2.15%)	954 (2.21%)	13 (0.69%)	
General pediatric surgery, *n* (%)	874 (1.94%)	870 (2.02%)	4 (0.21%)	
Gastric surgery, *n* (%)	832 (1.85%)	819 (1.90%)	13 (0.69%)	
Otolaryngology, *n* (%)	720 (1.60%)	719 (1.67%)	1 (0.05%)	
Orthopedics, *n* (%)	679 (1.51%)	678 (1.57%)	1 (0.05%)	
Neurology, *n* (%)	668 (1.48%)	649 (1.50%)	19 (1.01%)	
Pediatric nephrology, *n* (%)	649 (1.44%)	645 (1.50%)	4 (0.21%)	
Chest Surgery, *n* (%)	563 (1.25%)	546 (1.27%)	17 (0.9%)	
Pediatric neurosurgery, *n* (%)	502 (1.12%)	496 (1.15%)	6 (0.32%)	
Pediatric neurology, *n* (%)	449 (1.00%)	445 (1.03%)	4 (0.21%)	

Only those medical services that received at least 1% of total income are shown.

^a^Chi-square test.

^*^Significant p-value.

The main conditions diagnosed at admission according to the ICD-10 group of patients hospitalized during the study period in general and grouped by the absence or presence of in-hospital death are listed in Table 5. The results highlight that the four main ICD-10 main conditions diagnosed at admission groups (approximately 70% total) were, in descending order, [C00-D48] neoplasms, 16,710/45,011 (37.12%); [I00–I99] diseases of the circulatory system 3,625/45,011 (8.05%); [N00-N99] diseases of the genitourinary system 4,773/45,011 (10.60%); and [Q00-Q99] congenital malformations, deformities and chromosomal abnormalities 5,125/45,011 (11.39%). In addition, an association between the group with the main condition diagnosed at admission according to ICD-10 group and the absence or presence of in-hospital death was identified (*p* < 0.001).

**Table 5:** Main conditions diagnosed at admission according to the ICD-10 group of patients hospitalized in the selected hospital from 2007 to 2016.

**Variable**	**Overall (*n* = 45,011)**	**In-hospital death (–) (*n* = 43,132)**	**In-hospital death (+) (*n* = 1,879)**	**Intergroup comparison**
**Main condition diagnosed at admission according to ICD-10 group**				*p* < 0.001^a*^
I.- A00-B99 Certain infectious and parasitic diseases, *n* (%)	443 (0.98%)	420 (0.97%)	23 (1.22%)	
II.- C00-D48 Neoplasms, *n* (%)	16,710 (37.12%)	15,817 (36.67%)	893 (47.53%)	
III.- D50-D89 Diseases of the blood and hematopoietic organs and other disorders that affect the mechanism of immunity, *n* (%)	811 (1.80%)	775 (1.80%)	36 (1.92%)	
IV.- E00-E90 Endocrine nutritional and metabolic diseases, *n* (%)	1,021 (2.27%)	1,004 (2.33%)	17 (0.90%)	
V.- F00-F99 Mental and behavioral disorders, *n* (%)	84 (0.19%)	84 (0.19%)	0 (0.00%)	
VI.- G00-G99 Diseases of the nervous system, *n* (%)	1,670 (3.71%)	1,625 (3.77%)	45 (2.39%)	
VII.- H00-H59 Diseases of the eye and its annexes, *n* (%)	111 (0.25%)	111 (0.26%)	0 (0.00%)	
VIII.- H60-H95 Diseases of the ear and mastoid process, *n* (%)	374 (0.83%)	373 (0.86%)	1 (0.05%)	
IX.- I00-I99 Diseases of the circulatory system, *n* (%)	3,625 (8.05%)	3,315 (7.69%)	310 (16.50%)	
X.- J00-J99 Diseases of the respiratory system, *n* (%)	1,268 (2.82%)	1,192 (2.76%)	76 (4.04%)	
XI.- K00-K93 Diseases of the digestive system, *n* (%)	2,764 (6.14%)	2,636 (6.11%)	128 (6.81%)	
XII.- L00-L99 Diseases of the skin and subcutaneous tissue, *n* (%)	172 (0.38%)	165 (0.38%)	7 (0.37%)	
XIII.- M00-M99 Diseases of the musculoskeletal system and connective tissue, *n* (%)	2,729 (6.06%)	2,695 (6.25%)	34 (1.81%)	
XIV.- N00-N99 Diseases of the genitourinary system, *n* (%)	4,773 (10.60%)	4,715 (10.93%)	58 (3.09%)	
XV.- O00-O99 Pregnancy, childbirth and puerperium, *n* (%)	1 (0.00%)	1 (0.00%)	0 (0.00%)	
XVI.- P00-P96 Certain conditions originating in the perinatal period, *n* (%)	68 (0.15%)	66 (0.15%)	2 (0.11%)	
XVII.- Q00-Q99 Congenital malformations, deformities and chromosomal abnormalities, *n* (%)	5,125 (11.39%)	4,935 (11.44%)	190 (10.11%)	
XVIII.- R00-R99 Symptoms, signs and abnormal clinical findings and Laboratory, not elsewhere classified, *n* (%)	233 (0.52%)	211 (0.49%)	22 (1.17%)	
XIX.- S00-T98 Trauma, poisoning and some other consequences of external cause, *n* (%)	1,736 (3.86%)	1,710 (3.96%)	26 (1.38%)	
XX.- V01-Y98 Extreme causes of morbidity and mortality, *n* (%)	0 (0.00%)	0 (0.00%)	0 (0.00%)	
XXI.- Z00-Z99 Factors that influence the state of health and contact with health services, *n* (%)	1,293 (2.87%)	1,282 (2.97%)	11 (0.59%)	
XXII.- U00-U99 Codes for special situations, *n* (%)	0 (0.00%)	0 (0.00%)	0 (0.00%)	

^a^Chi-square test.

^*^Significant *p*-value.

The characteristics of the conditions of the patients hospitalized during the study period in general and grouped by the absence or presence of in-hospital death are listed in Table 6. In all hospitalized patients, the main condition diagnosed at admission was equal to the initial preadmission reference diagnosis at 37,412/45,011 (83.12%). Similarly, there was equality in ICD-10 groups of the main condition diagnosed at admission and the baseline diagnosis at 38,295/45,011 (85.08%). A total of 35,753/45,011 (79.43%) of patients did not present with comorbidities. An intergroup comparison using the Mann–Whitney *U* test revealed that the number of comorbidities (1.07 vs. 0.33; *p* < 0.001) was significantly higher in patients who died than in those who did not. Additionally, in the chi-square test, associations between the variable presence or absence of in-hospital death and the following variables were identified: equality in the main condition diagnosed at admission and the initial reference diagnosis, in general and grouped by ICD-10, as well as the presence of comorbidities (*p* < 0.001 in all cases).

**Table 6:** Characteristics of the conditions of hospitalized patients in the selected hospital from 2007 to 2016.

**Variable**	**Overall (*n* = 45,011)**	**In-hospital death (–) (*n* = 43,132)**	**In-hospital death (+) (*n* = 1,879)**	**Intergroup comparison**
Main condition diagnosed at admission equal to the initial preadmission reference diagnosis, *n* (%)	37,412 (83.12%)	35,957 (83.37%)	1,455 (77.43%)	*p* < 0.001^b*^
Equality between ICD-10 groups of the main condition diagnosed at admission and the initial preadmission reference diagnosis, *n* (%)	38,295 (85.08%)	36,774 (85.26%)	1,521 (80.95%)	*p* < 0.001^b*^
Number of additional conditions (comorbidities)	0.36 (0.88)	0.33 (0.83)	1.07 (1.42)	*p* < 0.001^a*^
Additional conditions (comorbidities) grouped				*p* < 0.001^b^^*^
No additional conditions	35,753 (79.43%)	34,814 (80.72%)	939 (49.97%)	
1 additional condition	5,367 (11.92%)	4,961 (11.5%)	406 (21.61%)	
2 additional conditions	2,080 (4.62%)	1,843 (4.27%)	237 (12.61%)	
3 additional conditions	983 (2.18%)	836 (1.94%)	147 (7.82%)	
4 additional conditions	450 (1%)	367 (0.85%)	83 (4.42%)	
5 additional conditions	239 (0.53%)	195 (0.45%)	44 (2.34%)	
6 additional conditions	139 (0.31%)	116 (0.27%)	23 (1.22%)	

Unless otherwise indicated, data are presented as the mean (standard deviation).

^a^Mann–Whitney *U* test.

^b^Chi-square test.

^*^Significant *p*-value.

### 3.3 Post-admission characteristics

In general, the patients underwent a mean (±standard deviation) of 1.08 ± 0.65 procedures (diagnostic, surgical or therapeutic). The mean ± standard deviation of the number of days of hospital stay was 6.25 ± 10.8; 28,223/45,011 (62.70%) had a stay of > 48 h, 12,293/45,011 (27.31%) had a stay of ≥7 days, and 4,706/45,011 (10.46%) had a stay of ≥14 days. Notably, 1,816/45,011 (4.03%) patients developed nosocomial infections; 1,454/43,132 (3.37%) patients presented with infection and did not die, and 362/1,879 (19.27%) patients presented with infection and died. In addition, in 44,646/45,011 (99.19%) of the patients, the medical service at discharge was equal to that at admission.

The post-admission characteristics of the patients hospitalized during the study period in general and grouped by the absence or presence of in-hospital death are detailed in Table 7. On the basis of the intergroup comparison by means of the Mann-Whitney U test, the number of procedures (1.11 vs. 1.08; *p* < 0.001), the number of procedures under general anesthesia (0.54 vs. 0.53; *p* = 0.002) and the length of stay in days (11.74 vs. 6.01; *p* < 0.001) were significantly higher in those patients who died. Additionally, analysis with the chi-square test revealed an association between the presence or absence of in-hospital death and the following variables: stay >48 h, stay ≥7 days, stay ≥14 days, presence of nosocomial infection, day of the week of discharge (*p* < 0.001 in all cases), month of discharge (*p* = 0.064), and equality between the service of admission and that of discharge (*p* = 0.086).

**Table 7:** Post-admission characteristics of hospitalized patients in the selected hospital from 2007 to 2016.

**Variable**	**Overall (*n* = 45,011)**	**In-hospital death (–) (*n* = 43,132)**	**In-hospital death (+) (*n* = 1,879)**	**Intergroup comparison**
Number of procedures (diagnostic, surgical or therapeutic)	1.08 (0.65)	1.08 (0.63)	1.11 (1.02)	*p* < 0.001^a^^*^
Number of procedures under general anesthesia	0.53 (0.70)	0.53 (0.69)	0.54 (0.85)	*p* = 0.002^a^^*^
Days of stay	6.25 (10.8)	6.01 (10.59)	11.74 (13.77)	*p* < 0.001^a^^*^
Hospital stay> 48 h, *n* (%)	28,223 (62.70%)	26,810 (62.16%)	1,413 (75.20%)	*p* < 0.001^b^^*^
Hospital stay ≥7 days, *n* (%)	12,293 (27.31%)	11,292 (26.18%)	1,001 (53.27%)	*p* < 0.001^b^^*^
Hospital stay ≥14 days, *n* (%)	4,706 (10.46%)	4,144 (9.61%)	562 (29.91%)	*p* < 0.001^b^^*^
Nosocomial infection, *n* (%)	1,816 (4.03%)	1,454 (3.37%)	362 (19.27%)	*p* < 0.001^b^^*^
Day of the week of discharge				*p* < 0.001^b^^*^
Monday, *n* (%)	6,249 (13.88%)	5,963 (13.83%)	286 (15.22%)	
Tuesday, *n* (%)	6,975 (15.50%)	6,682 (15.49%)	293 (15.59%)	
Wednesday, *n* (%)	7,970 (17.71%)	7,721 (17.90%)	249 (13.25%)	
Thursday, *n* (%)	7,723 (17.16%)	7,417 (17.20%)	306 (16.29%)	
Friday, *n* (%)	10,673 (23.71%)	10,397 (24.11%)	276 (14.69%)	
Saturday, *n* (%)	3,826 (8.50%)	3,605 (8.36%)	221 (11.76%)	
Sunday, *n* (%)	1,595 (3.54%)	1,347 (3.12%)	248 (13.20%)	
Month of discharge				*p* = 0.064^b^
January, *n* (%)	3,100 (6.89%)	2,960 (6.86%)	140 (7.45%)	
February, *n* (%)	3,353 (7.45%)	3,197 (7.41%)	156 (8.30%)	
March, *n* (%)	3,794 (8.43%)	3,642 (8.44%)	152 (8.09%)	
April, *n* (%)	3,463 (7.69%)	3,304 (7.66%)	159 (8.46%)	
May, *n* (%)	3,666 (8.14%)	3,516 (8.15%)	150 (7.98%)	
June, *n* (%)	3,796 (8.43%)	3,636 (8.43%)	160 (8.52%)	
July, *n* (%)	4,082 (9.07%)	3,928 (9.11%)	154 (8.20%)	
August, *n* (%)	4,052 (9.00%)	3,902 (9.05%)	150 (7.98%)	
September, *n* (%)	3,900 (8.66%)	3,764 (8.73%)	136 (7.24%)	
October, *n* (%)	4,295 (9.54%)	4,124 (9.56%)	171 (9.10%)	
November, *n* (%)	3,753 (8.34%)	3,571 (8.28%)	182 (9.69%)	
December, *n* (%)	3,757 (8.35%)	3,588 (8.32%)	169 (8.99%)	
Medical service at discharge equal to admission, *n* (%)	44,646 (99.19%)	42,789 (99.2%)	1,857 (98.83%)	*p* = 0.086^b^

Unless otherwise indicated, data are given as the mean (standard deviation).

^a^Mann–Whitney *U* test.

^b^Chi-square test.

^*^Significant *p*-value.

### 3.4 Model fitting and performance evaluation

#### 3.4.1 Modeling approach

**Figure 1:**
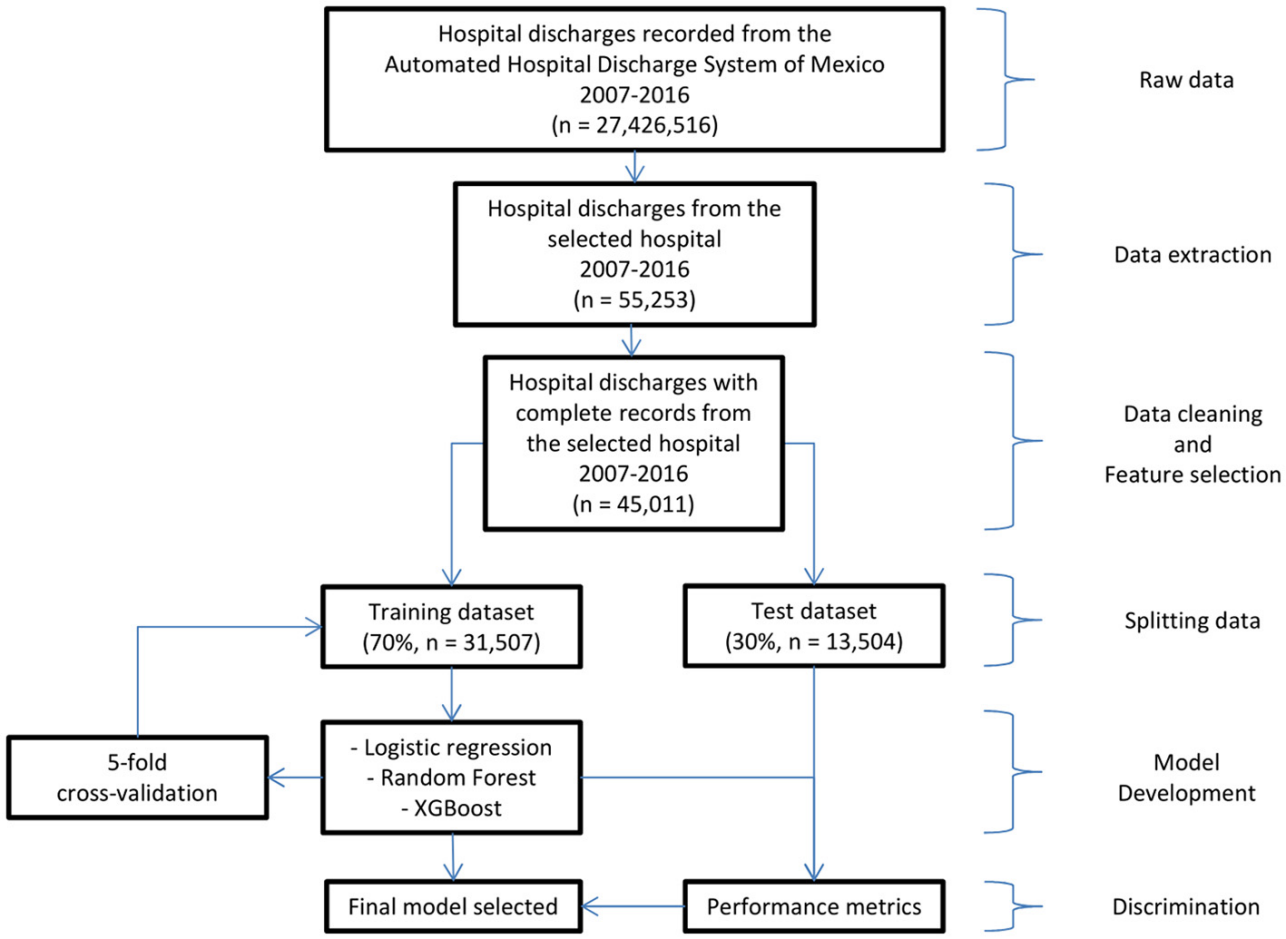
Workflow diagram for model development and evaluation.

To fit the machine learning models, the dataset (*n* = 45,011; 1,879 in-hospital deaths) was randomly divided into a training set (70% of the data, *n* = 31,507; 1,293 in-hospital deaths) and a test set (30% of the data, *n* = 13,504; 586 in-hospital deaths). The scheme for fitting the in-hospital death predictive models is shown in Figure 1. The training set was divided into five parts, with which each model was cross-validated.

#### 3.4.2 Model training

Based on the training set, the best fitted multivariate model (higher AUC) of each type (logistic regression, random, forest and XGBoost) that resulted from considering the 16 preadmission characteristics of the patients as predictor variables are detailed below. For the case of logistic regression, the best model was the one that included the 16 characteristics, which generated a Nagelkerke *R*^2^ = 0.443, a *p* = 0.447 associated with the Hosmer and Lemshov test and an AUC = 0.9237 with a CI of 95% (0.9162–0.9312). In the case of the random forest, the best model included the 16 characteristics and considered the following parameters: the number of characteristics used to divide each node (mtry = 4) and the number of trees (ntree = 1000), which generated an AUC = 0.9892 with a 95% CI (0.9853–0.9931). For XGBoost, the best model included the 16 characteristics and considered the following parameters: number of iterations (nrounds = 1,000), maximum depth per tree (max.depth = 6) and learning rate (eta = 0.01), which generated an AUC = 0.9563 with a 95% CI (0.9512–0.9614).

#### 3.4.3 Variable importance

**Figure 2:**
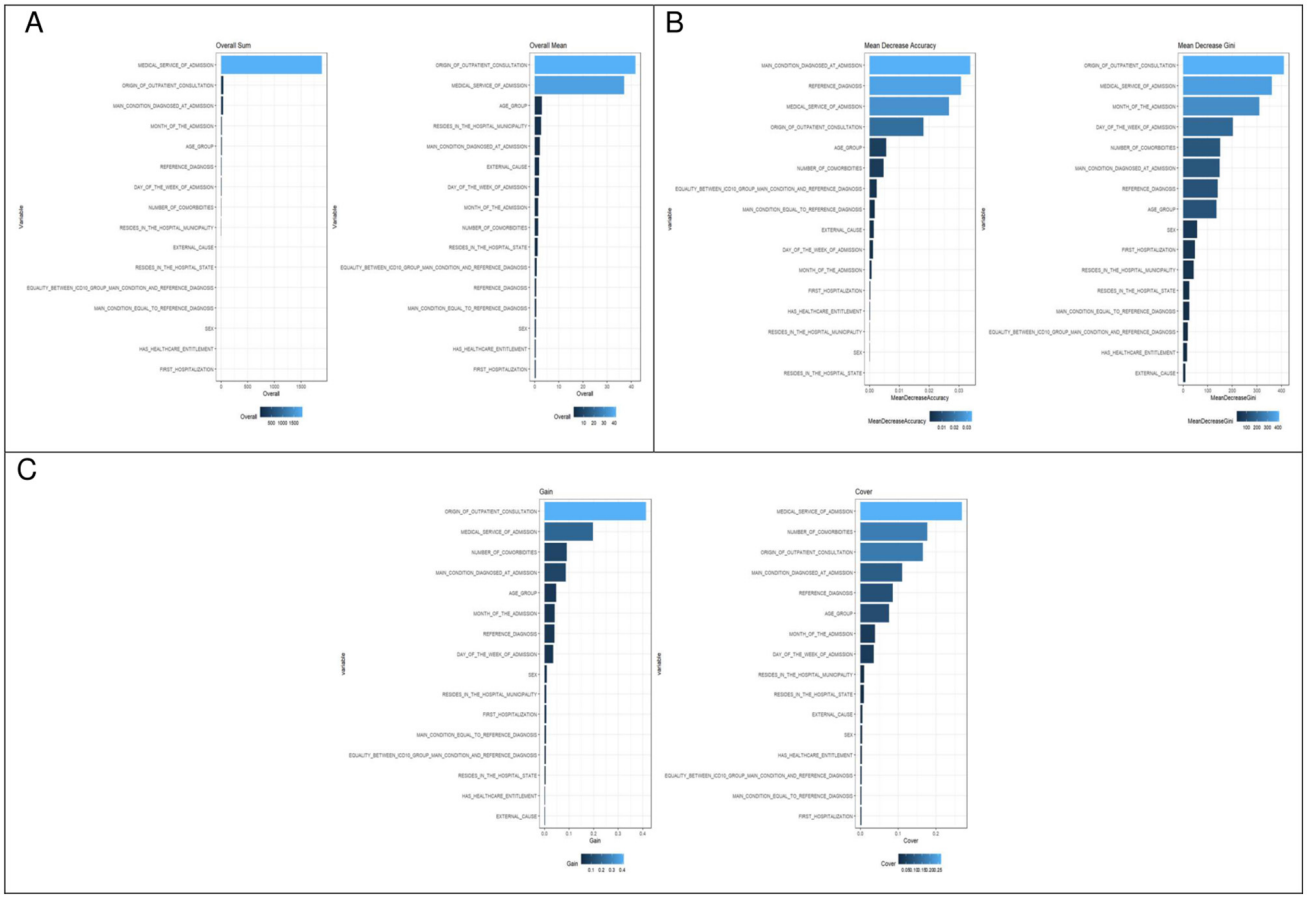
Variable importance for predicting in-hospital death across modeling approaches. **(A)** Logistic regression. **(B)** Random forest. **(C)** XGBoost.

The importance of each of the preadmission variables that predict in-hospital death in descending order for each model is shown in Figure 2. In the case of logistic regression, the most important variables are the origin of the hospital outpatient consultation and the medical service that performed the admission. For the random forest model, the variables that are the most important are the origin of the hospital outpatient consultation, the medical service that performed the admission, the month of admission and the day of the week of admission. In the XGBoost model, the most important variables are the medical service that performed the admission, the number of conditions, the origin of the hospital outpatient consultation and the main condition diagnosed upon admission according to the ICD-10.

#### 3.4.4 Model comparison

**Figure 3:**
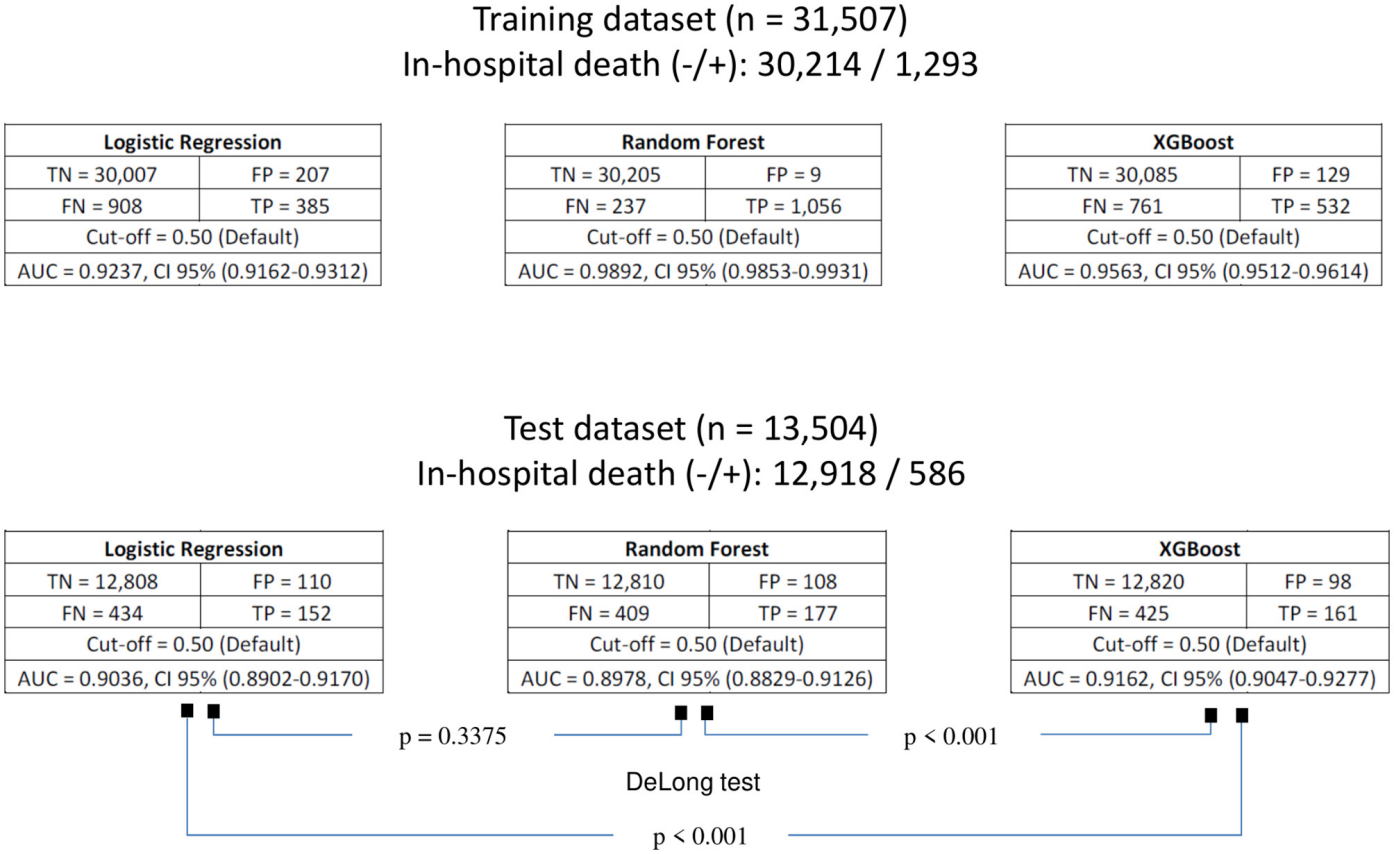
Confusion matrices of the fitted models for predicting in-hospital death for both training and test datasets. TN, true negative; FP, false positive; FN, false negative; TP, true positive; AUC, area under curve; CI, confidence interval.

**Figure 4:**
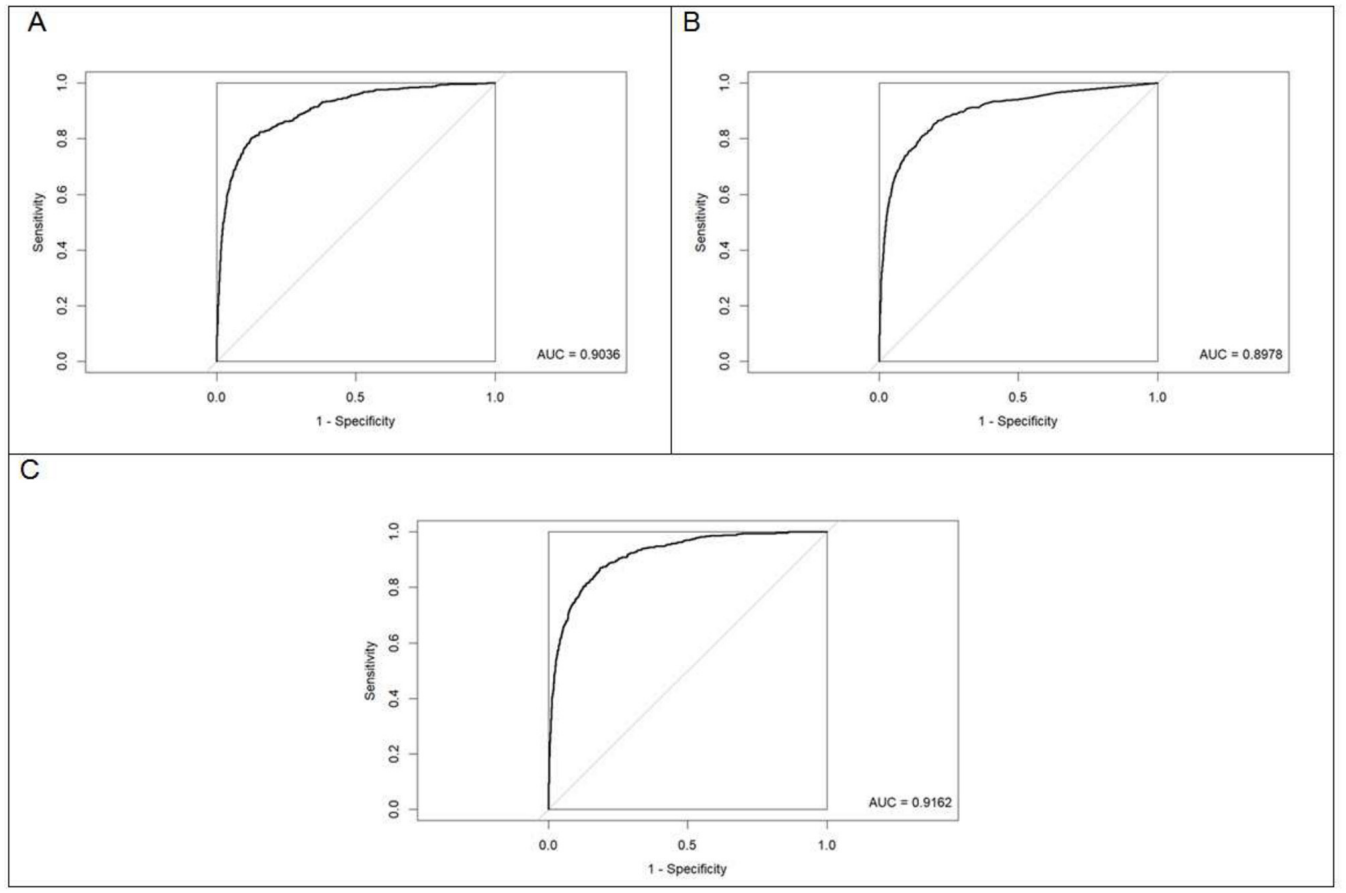
Receiver operating characteristic (ROC) curves for the fitted models evaluated on the test dataset. **(A)** Logistic regression. **(B)** Random forest. **(C)** XGBoost. The diagonal line indicates the performance of a random classifier (reference line, AUC = 0.50).

The confusion matrices, as well as the areas under the curve (AUCs), with their respective 95% confidence intervals for each of the models fitted with preadmission variables for the prediction of in-hospital death on the basis of the SAEH-HRAEB 2007–2016 data, both in the training set and in the test set is shown in Figure 3. In all cases, a cutoff point equal to 0.5 was considered. Notably, on the basis of the test set and when two-to-two comparisons of the AUCs (using Delong tests) between the three models were performed, a higher AUC was detected in the model built with XGBoost AUC = 0.9162 (0.9047–0.9277) than in those constructed using logistic regression AUC = 0.9036 (0.8902–0.9170) and random forest AUC = 0.8978 (0.8829–0.9126) (*p*-value < 0.001 in both cases), which suggests that the performance of the constructed model via XGBoost is better. In addition, the ROC curves constructed based on the test set for each of the models (logistic regression, random forest and XGBoost) are shown in Figure 4.

#### 3.4.5 Model performance metrics

Based on the test set, when exploring the optimal cutoff points in the construction of ROC curves (criterion of the point closest to [0,1] of the ROC curve), as shown in Table 8, these cutoff points are detailed as follows as the efficiency metrics of the models to predict in-hospital death considering 16 preadmission variables. The logistic regression model generated an AUC = 0.904 (0.890–0.917), a cutoff point equal to 0.040, a sensitivity of 80.5%, a specificity of 87.2% and a balanced efficiency of 83.9%. The random forest model generated an AUC = 0.898 (0.883–0.912), a cutoff point equal to 0.037, a sensitivity of 85.3%, a specificity of 80.3% and a balanced efficiency of 82.8%. The XGBoost model generated an AUC = 0.916 (0.905–0.928), a cutoff point equal to 0.023, a sensitivity of 87.0%, a specificity of 81.3% and a balanced efficiency of 84.2%. The DeLong test revealed that the area under the curve of the XGBoost model was greater than that of the logistic regression and random forest models (*p*-value < 0.001 in both cases).

**Table 8:** Performance metrics of the machine learning models fitted for the prediction of in-hospital death.

**Data**	**Model type**	**Number of preadmission variables**	**Cut point criteria**	**Cut off**	**Specificity**	**Sensitivity**	**Balanced accuracy**	**Accuracy**	**NPV**	**PPV**	**Precision**	**Recall**	**F1 score**	**AUC**
Training dataset 2007–2016 *n* = 31,507 Death (–/+) 30,214/1,293	LR	16	C	0.037	0.864	0.839	0.852	0.863	0.992	0.209	0.209	0.839	0.335	0.924 (0.916–0.931)
	RF	16	C	0.134	0.974	0.959	0.967	0.974	0.998	0.614	0.614	0.959	0.749	0.989 (0.985–0.993)
	XGBoost	16	C	0.042	0.884	0.887	0.886	0.884	0.995	0.247	0.247	0.887	0.386	0.956 (0.951–0.961)
Test dataset 2007–2016 *n* = 13,504 Death (–/+) 12,918/586	LR	16	C	0.040	0.872	0.805	0.839	0.869	0.990	0.221	0.221	0.805	0.347	0.904 (0.890–0.917)
	RF	16	C	0.037	0.803	0.853	0.828	0.805	0.992	0.164	0.164	0.853	0.276	0.898 (0.883–0.912)
	XGBoost	16	C	0.023	0.813	0.870	0.842	0.815	0.993	0.174	0.174	0.870	0.290	0.916 (0.905–0.928)

LR, Logistic Regression; RF, Random Forest; XGBoost, Extreme Gradient Boosting; C, Closest; NPV, Negative Predictive Value; PPV, Positive Predictive Value; AUC, Area Under the Curve.

**Figure 5:**
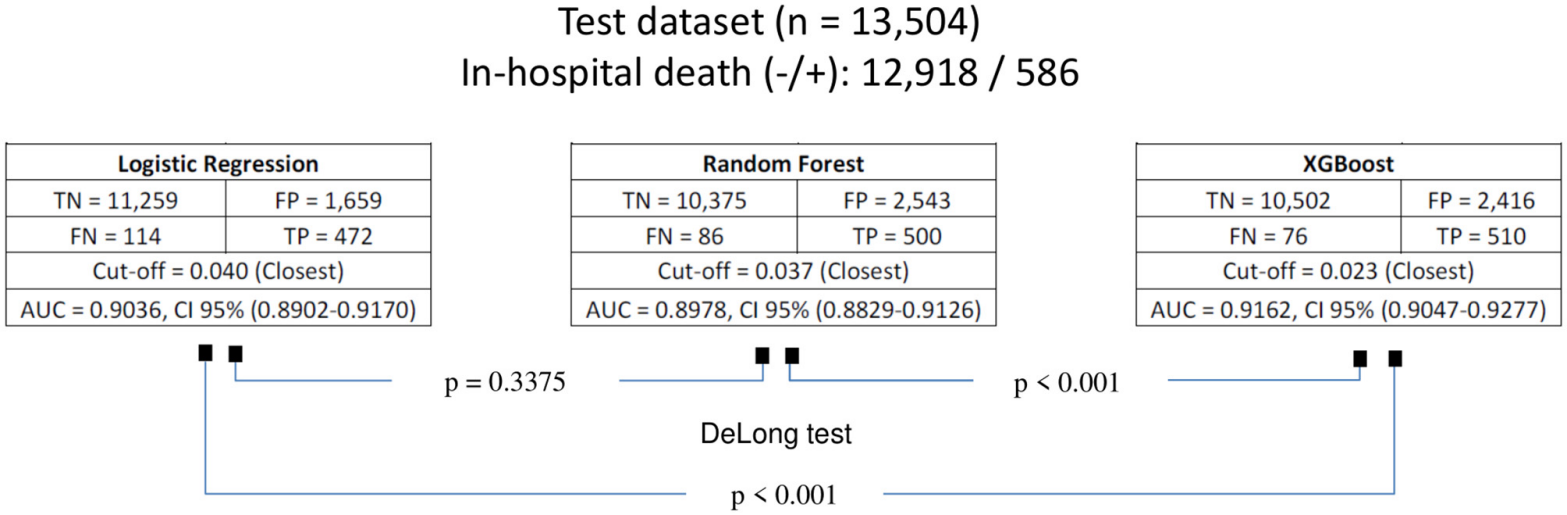
Confusion matrices of the models for predicting in-hospital death, evaluated on the test dataset using the optimal cut-off points, defined by the closest to [0,1] criterion on the ROC curve. TN, true negative; FP, false positive; FN, false negative; TP, true positive; AUC, area under curve; CI, confidence interval.

Finally, the confusion matrices for all models evaluated on the test set, along with their respective optimal cut-off points, are presented in Figure 5. Notably, for the best-performing model for predicting in-hospital death was XGBoost. When evaluated on the test set (*n* = 13,504; 586 in-hospital deaths), this model identified 10,502 true negatives, 76 false negatives, 2,416 false positives, and 510 true positives. This result in a ratio of 4.7 to 1 for patients predicted to die in-hospital who ultimately survived, compared those who actually died.

## 4 Discussion

Hospital mortality is a multidimensional indicator widely used to evaluate the quality and effectiveness of medical care (6, 7). Identifying the factors associated with in-hospital death that facilitate the construction of robust predictive models is essential. The current computing power allows the implementation of complex algorithms that exceed the capabilities of traditional logistic regression. Among these predictive models, approaches such as random forest (30) and XGBoost (34), stand out, as they are able to identify complex patterns between the data.

The identification of factors with high prognostic value for in-hospital death is crucial in the hospital setting since it provides the opportunity to carry out closer monitoring of patients at risk, helping to reduce the frequency of adverse events and therefore improving the quality of hospital care. This study seeks to adjust machine learning models to predict and identify the preadmission factors with the highest prognostic value for in-hospital death.

### 4.1 In-hospital mortality and preadmission characteristics

In our study, in which the hospital discharge records of 45,011 patients in a third-level Mexican hospital during its first 10 years of operation were analyzed, an in-hospital mortality of 4.17% was observed. This figure is similar to that reported by Le Guen and Tobin (53), who reported a mortality of 3.62% in 44,297 patients discharged from an Australian tertiary care hospital over a period of 5 years. However, it is higher than the 2.15% registered nationally in Mexican hospitals in the same 10-year period of our study (3). This could be attributed to the greater complexity of the pathologies treated in tertiary care hospitals.

Additionally, the mean age of the patients who died in the hospital was significantly higher than that of patients who survived, a finding that is consistent with that reported by Clark et al. (24) in a multicenter study in which 10,743 hospital admissions were considered and patients younger than 60 years were reported to have a risk of in-hospital death that was up to three times lower than that of patients older than 60 years. Likewise, a significant association was found between in-hospital death and the day of the week of admission, which is consistent with the findings of Mohammed et al. (16) in a multicenter study that included approximately 1.5 million hospital admissions, hospital admission on the weekend was a risk factor for in-hospital death, and the risk was more pronounced in elective admissions than in emergency admissions.

On the other hand, the month of admission was also associated with in-hospital death. This phenomenon could be explained in terms of seasonal variability in mortality, as reported by Achebak et al. (54), who, in their analysis of approximately 1.7 million hospital admissions, reported that although the number of hospitalizations for respiratory diseases increases during cold months, in-hospital mortality peaks during months of high temperatures, probably because of the additional effect of heat on the vulnerability of patients with chronic respiratory diseases.

Regarding other associated factors, the first hospitalization, coming from the outpatient clinic, as well as the equality between the main condition diagnosed at admission and the reference diagnosis, was associated with lower in-hospital mortality. In this sense, in 83.12% of the 45,011 hospital discharges, the main condition diagnosed at admission coincided with the reference diagnosis.

In contrast, factors such as the reference diagnosis, the main condition diagnosed at admission, the medical service that performed the admission, the presence of comorbidities and a higher number of comorbidities were associated with higher in-hospital mortality. Among the 45,011 hospital discharges, the main ICD-10 diagnostic groups at admission were neoplasms (37.12%), genitourinary diseases (10.60%), congenital malformations (11.39%) and diseases of the circulatory system (8.05%), accounting for approximately 70% of the total. In addition, approximately half of the 1,879 in-hospital deaths were patients admitted by one of the following five services: oncology (21.18%), hematology (9.53%), internal medicine (9.21%), neurosurgery (4.79%) and cardiology (4.58%). Notably, 79.43% of the 45,011 hospital discharges did not present comorbidities.

Our findings suggest that patients with in-hospital death tend to enter a worse clinical state and have less prior information about their medical condition, either because of a late diagnosis or the presence of a serious acute illness. In addition, those without contact prior to outpatient care do not have a known clinical history or a history of response to previous treatments, which makes their management difficult. This clinical context limits the capacity for therapeutic response and increases the risk of fatal outcomes. These findings are consistent with those proposed by models such as the HOMR (Hospital Patient One-year Mortality Risk), built on the basis of 640,022 hospital admissions (55), which identifies the admission diagnosis, the admission medical service and the number of comorbidities as key predictors of hospital mortality (55).

### 4.2 Model performance and interpretability

To fit the three types of supervised machine learning models (logistic, random forest and XGBoost) considered for predicting in-hospital death on the basis of preadmission characteristics, our 45,011 hospital discharges were randomly divided into two subsets: 70% for training and 30% for testing. This proportion has been used in studies such as the one by Wen et al. (56), in which eight types of machine learning models were evaluated to predict 28-day mortality in patients with sepsis. In other studies, such as that by Cao et al. (57), an 80% ratio for training was used, and a 20% ratio was used for testing when an XGBoost model was developed to predict in-hospital mortality in a cohort of 545,388 patients with severe traumatic brain injury. These variations reflect that the data partition can be adapted to the sample size, but in all cases, a balance between model fit and generalizability is sought.

In addition, the inclusion of multiple models allows us to compare not only their precision but also their ability to identify clinically relevant factors with prognostic value. In our study, machine learning methods were shown to be effective in predicting in-hospital mortality using only data available at the time of admission.

Through the test set and the implementation of the DeLong test, the XGBoost model outperformed logistic regression (AUC = 0.9036, 95% CI: 0.8902–0.9170) and random forest (AUC = 0.8978, 95% CI: 0.8829–0.9126), with a *p*-value < 0.001 in both cases.

Overfitting, which arises when a model fits the training data too closely and loses generalizability (58), was evident in the Random Forest model, with near-perfect discrimination on the training set (AUC = 0.989) but reduced discriminatory ability on the test set (AUC = 0.898). In contrast, XGBoost showed only a mild decrease in discrimination for training (AUC = 0.956) to test data (AUC = 0.916), which can be attributed to the explicit regularization mechanisms (e.g., learning rate and depth control) that limit model complexity.

The XGBoost model reached an AUC = 0.916, 95% CI: 0.905–0.928, with an optimal cutoff point of 0.023 (according to the criterion of the point closest to [0.1] on the ROC curve), a sensitivity of 87.0%, a specificity of 81.3%, and a balanced efficiency of 84.2%. These values indicate that the model has high discrimination and an adequate balance between false positives and negatives in real clinical conditions.

With respect to interpretability, the models identified variables with greater predictive importance. In logistic regression, the origin of the outpatient consultation of the hospital and the medical service that performed the admission stood out. In the random forest model, there was a coincidence with logistic regression, but the month of admission and the day of the week of admission were added. Finally, in XGBoost, the most relevant variables were medical service that performed the admission, number of conditions, origin of the outpatient consultation of the Hospital, the main condition diagnosed at admission according to the ICD-10, age, month of admission, and day of the week of admission. These differences in the variables with greater predictive importance highlight the ability of complex models to detect nonlinear interactions and hidden relationships between predictors.

Notably, “medical service that performed the admission” emerged as the single strongest predictor of in-hospital death in the XGBoost model, likely serving as a proxy for critical clinical factors such as disease severity, case-mix complexity, and variations in admission or treatment practices. In our cohort, oncology (21.18%, 398/1,879), hematology (9.53%, 179/1,879), and internal medicine (9.21%, 173/1,879) contributed the largest shares of in-hospital deaths, reflecting known high-risk patient populations: oncology patients frequently present with advanced or metastatic disease (53), hematology patients mainly with various types of leukemia receiving chemotherapy regimens that carry high treatment-related risks including infections and complications (59), and internal medicine patients often present with complex or undifferentiated conditions, which may delay definitive diagnosis and timely intervention (60). The predictive prominence of this variable thus captures critical aspects of patient complexity at admission, highlighting its practical value for early risk stratification and targeted preventive interventions.

Our best fitted model to predict in-hospital death results with XGBoost AUC = 0.916 (0.905–0.928), when evaluated in the test set (*n* = 13,504; 586 in-hospital deaths), allows us to identify 10,502 true negatives, 76 false-negatives, 2,416 false positives and 510 true positives, which generates a ratio of 4.7 to 1 of false positives for every true positive. Although this alert rate may appear high, in the clinical setting, it could be considered reasonable since it is based solely on preadmission characteristics and allows timely detection of patients at risk, facilitating early preventive interventions.

Similar findings were reported by Soffer et al. (61). In particular, when XGBoost was applied to a cohort of 118,262 hospitalized patients (6,311 in-hospital deaths), an AUC of 0.924 (95% CI: 0.917–0.930) and a ratio of 5.9 to 1 of false positives for each true positive were achieved on the basis of preadmission characteristics and comorbidities, together with data on laboratory tests and initial treatment. This concordance supports the clinical potential of the XGBoost model as a useful tool for identifying patients at high risk of in-hospital mortality, especially when it is based on patient data available at hospital admission.

In the clinical practice, the integration of the model into the electronic health record as a decision-support alert would enable admitting physicians to identify high-risk patients early (62), guiding timely preventive actions while minimizing alert fatigue and clinician burden. Rather than functioning as a hard stop, the model could serve as a first-line screening tool, prompting secondary evaluations, such as targeted laboratory tests, imaging studies, or enhanced monitoring, to refine risk stratification. This approach aligns with prior studies demonstrating the practical application of AI-based risk prediction systems in daily medical practice (63–65), where models produce probabilities or scores that inform, rather than dictate, clinical decision-making.

In summary, our results show that the use of advanced algorithms such as XGBoost allows not only improvement of the accuracy of the prediction but also optimization of the identification of clinically relevant risk factors, offering a solid basis for the development of early warning systems for mortality in high-complexity hospitals.

### 4.3 Limitations, strengths, and future directions

Our study has certain limitations. First, since it is a cross-sectional study, it does not allow us to infer causality. Second, the results are based on a single-center study that uses data from a secondary base, which may contain some inherent capture errors at the source. Third, predictive models of in-hospital death were not constructed on the basis of medical services at admission or specific pathologies. Fourth, 18.54% of discharge records were excluded due to incomplete data; although most variables were nearly complete and missingness was not systematically associated with patient characteristics or adverse outcomes, the excluded records included only 146 in-hospital deaths (1.43%, 146/10,242), which may still introduce potential selection bias and affect model performance and variable importance. Finally, the lack of a preadmission biochemical or imaging characterization of patients limits the possibility of exploring potential associations and the predictive capacity of various biomarkers that could generate more robust models to predict in-hospital mortality.

Despite these limitations, our study has notable strengths, such as the evaluation of preadmission characteristics in a large sample (n = 45,011) of patients corresponding to a long period of time and the first 10 years of operation at a third-level care hospital. In addition, it was not restricted to the use of logistic regression but rather incorporated advanced machine learning techniques capable of identifying complex relationships between the predictor variables. This allowed identification of the factors that induce a greater propensity to present in-hospital death, improving the predictive capacity and understanding of the associated risks.

Finally, given the limitations identified, it is recommended that multicenter and longitudinal studies that include biochemical and imaging characteristics from hospital admission be carried out. In addition, it would be useful to develop specific models for different admission medical services, such as medical, surgical, and pediatric, including high-risk services like oncology and hematology, as well as specific pathologies. This approach would allow for greater precision in the identification of patients at high risk of in-hospital death, ideally externally validated and providing more context-specific estimates.

## 5 Conclusion

The XGBoost model, owing to its ability to capture complex patterns, makes it possible to identify patients with a higher risk of in-hospital death from the data available at hospital admission. This study provides a support tool for clinical decision-making and helps in the early identification of patients who require closer monitoring and follow-up during their hospital stay. This approach not only helps prioritize resources efficiently but also optimizes intervention strategies, significantly contributing to reducing in-hospital mortality and ultimately improving quality and comprehensive care in the hospital setting.

## Data Availability

Publicly available datasets were analyzed in this study. This data can be found here: http://www.dgis.salud.gob.mx/contenidos/basesdedatos/da_egresoshosp_gobmx.html.
